# Supine Traction vs Fulcrum Bending Radiographs in Preoperative Imaging of Scoliosis Patients Treated With Magnetically Controlled Growing Rods (MCGR) – which Technique is Better to Predict Surgical Correction of the Main Curve?

**DOI:** 10.1177/21925682241299339

**Published:** 2025-01-10

**Authors:** Stefan Hemmer, Raphael Trefzer, Tobias Renkawitz, Wojciech Pepke

**Affiliations:** 1 Department of Orthopaedics, 9144 University Clinic Heidelberg, Heidelberg, Germany

**Keywords:** traction, bending, scoliosis, magnetically controlled growing rods, curve correction, prediction, idiopathic, neuromuscular, juvenile, spine, level 4, retrospective cohort study

## Abstract

**Study Design:**

Retrospective Cohort Study.

**Objectives:**

Flexibility radiographs such as traction or bending radiographs are essential in preoperative imaging to assess for curve flexibility and to estimate the amount of operative correction in order to determine the type and length of instrumentation in growth-accompanying scoliosis treatment. Both traction and bending radiographs are controversially discussed in the literature. The predictability of flexibility radiographs of postoperative main curve correction specifically in patients treated with magnetically controlled growing rods (MCGR) has not yet been studied.

**Methods:**

Juvenile patients with idiopathic or neuromuscular scoliosis that were surgically treated with a primary MCGR implant with pedicle screw fixation between 2018-2022 were retrospectively registered. Patients that underwent prior spine surgery, with supine lying-down radiograph and patients with missing traction or bending radiographs available were excluded. Image analysis was conducted using Surgimap® software. For statistical analysis, *t* test and ANOVA analysis were used to compare the means between groups with a significance level set at *P* < 0.05.

**Results:**

A total of 50 patients, 34 diagnosed with idiopathic scoliosis (IS) and 16 diagnosed with neuromuscular scoliosis (NMS), were included. Globally, main curve Cobb angles were significantly higher in supine traction compared to fulcrum bending images (44.8° vs 39.6°; *P* < 0.001) and in the IS subgroup (42.4° vs 37.3°; *P* < 0.001). Compared to postoperative images, significant differences of supine traction but not fulcrum bending radiographs were detected in total (*P* < 0.001; *P* = 0.20) as well as IS (*P* < 0.001; *P* = 0.32) and NMS (*P* < 0.001; *P* = 0.44) subgroups. Fulcrum bending images displayed significantly higher flexibility rates (FR) and flexibility index (FI) compared to traction images in total (FR: 42.9 vs 35.2, *P* < 0.001; FI: 1.08 vs 1.58, *P* = 0.024) and the IS subgroup (FR: 44.2 vs 35.8, *P* < 0.001; FI: 1.19 vs 1.43, *P* = 0.033).

**Conclusions:**

Fulcrum bending radiographs showed better flexibility and prediction of operative main curve correction compared to supine traction radiographs in total and IS subgroup. Fulcrum bending might be more precise for predicting the postoperative main curve correction potential of primary MCGR surgery in IS patients.

## Background

The MCGR system was introduced a decade ago for growth accompanying treatment of severe juvenile scoliosis where conservative brace therapy fails, as an alternative for traditional growing rods that required frequent operative lengthening.^1^ Once implanted, the dual rod system can be distracted periodically through a noninvasive magnet distraction device in an outpatient setting until the patient reaches the adequate age for definitive posterior spinal fusion surgery (PSF).^2,3^ MCGR implantation results in substantial correction of the primary and secondary curve.^4^

Traction radiographs and bending radiographs are both used in preoperative imaging to assess for curve flexibility and to estimate the amount of correction that can be expected from operative instrumentation. However, both techniques are widely discussed in the literature and the utilization of specific flexibility assessments rather depends on in-house experience than scientific evidence.

Bending radiographs are widely used to determine curve flexibility and fusion levels in idiopathic scoliosis. Two main techniques of bending radiographs have been proposed: Active supine side bending radiographs (SBR)^5-7^ and fulcrum bending radiographs (FBR).^8-10^ SBR are considered as standard examination and are required for classification of curves.^11^ However, FBR were reported to reach higher rates of flexibility and better correlation with postoperative correction.^12-14^ Traction radiographs commonly play a role for large curves or neuromuscular scoliosis cases where FBR are more difficult to conduct. In former studies, SBR were found to display greater flexibility in curves <50°.^7,15^ However, greater curves showed more flexibility in supine traction radiographs.^15,16^ However, FBR was described as the best method to predict postoperative correction for posterior spinal fusion (PSF) in idiopathic scoliosis (IS).^17^

Regarding radiological outcomes specifically in MCGR surgery, the adequate method of flexibility assessment for prediction of the postoperative curve correction has not been investigated yet. Sufficient curve correction is necessary for successful MCGR treatment, as the correction can be maintained over time until patients undergo definitive spinal fusion but further correction by distraction of the system cannot be expected.^18^ Hence, individual preoperative preparation and planning including estimation of the surgical curve correction is essential.

In this study, we compared the supine traction radiograph (STR) and the fulcrum bending radiograph (FBR) technique regarding the predictability of postoperative main curve correction in a population of patients that received MCGR surgery. The purpose was to provide findings that can help surgeons to preoperatively estimate the main curve correction potential of MCGR implantation.

## Methods

### Patients

In this monocenter retrospective study, patients with scoliosis that were surgically treated with a primary MCGR implant with pedicle screw fixation between 2018-2022 were retrospectively analyzed. The indication for MCGR implantation was a Cobb-angle over 45° and skeletal immaturity with relevant expected growth, according to Sanders classification ≤ 3.^19^ Exclusion criteria were: Prior spine surgery, implant fixation other than pedicle screws and no preoperative STR and FBR available. All eligible patients that did not meet these exclusion criteria were included in the study.

Ethical approval was obtained from the Heidelberg University ethics committee (S-418/2022). It was opted to forego obtaining patient informed consent, as synonymized data were solely collected retrospectively from preexisting clinical data (baseline and radiographs) from the clinical routine work. Data were only analyzed by treating medical doctors who were authorized for access of the data.

### Data Acquisition and Management

**Figure 1.**
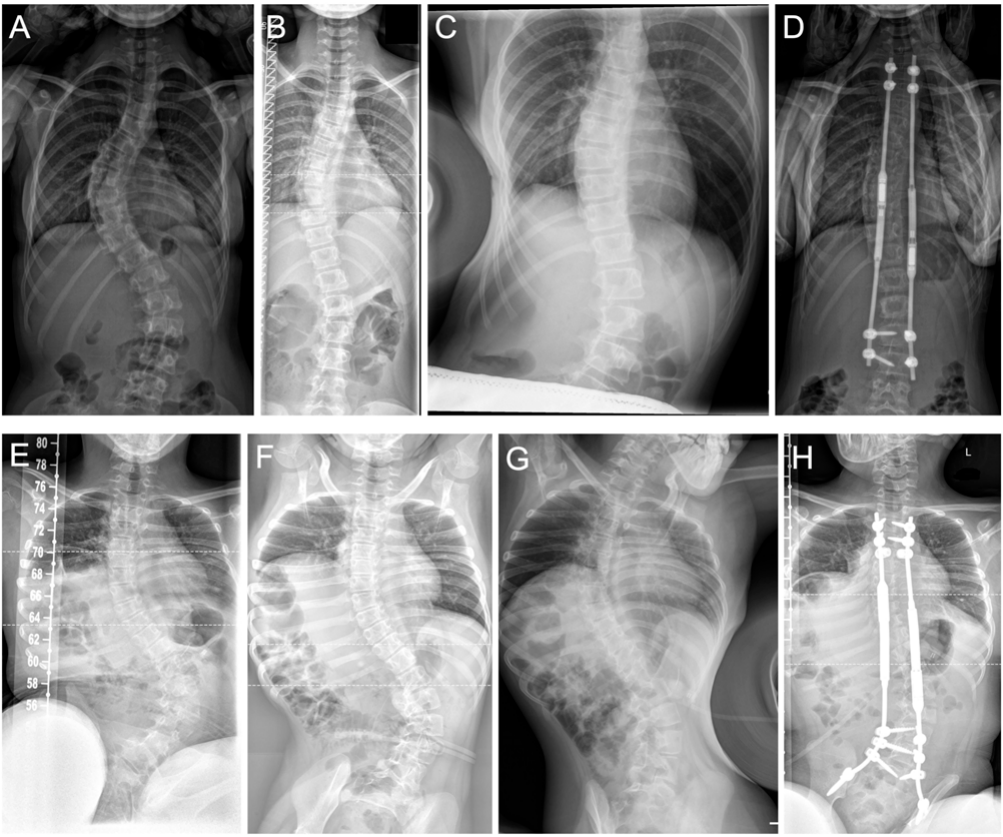
A. p. radiographs showing correction potential of the main curve. Upper panel: Patient suffering from IS: (A) Preoperative standing radiograph. (B) Preoperative supine traction radiograph. (C) Preoperative fulcrum bending radiograph. (D) Postoperative standing radiograph after primary MCGR-implantation. Lower panel: Nonambulatory patient suffering from NMS: (E) Preoperative sitting radiograph. (F) Preoperative supine traction radiograph. (G) Preoperative fulcrum bending radiograph. (H) Postoperative sitting radiograph after primary MCGR-implantation.

Baseline demographic data were obtained from patient records. Only radiographs that were obtained within clinical routine preoperative examinations were retrospectively acquired for image analysis. Prior to surgery, each patient underwent radiologic examination with standing AP and lateral and supine traction AP radiographs (STR) of the whole spine and fulcrum side bending radiographs FBR) of the main curve (Figure 1). STR were taken with the patient in supine position, and traction applied at the neck with a submandibular grip by an experienced medical doctor (Figure 1B, F). FBR were taken with the patient lying on their convex curve side with a fulcrum placed under the apex of the spinal curve. Patients were bend over the fulcrum in lateral position with the apex of the curve on the top of the fulcrum and the radiograph was taken in anteroposterior beam path (Figure 1C, G). The patient positioning was conducted by experienced radiological assistants. In our clinic, no cost difference exists between the 2 imaging options.

After surgery, standing AP and lateral radiographs of the whole spine were obtained. For nonambulatory NMS patients, preoperative and postoperative imaging was conducted in a sitting position. Measurements of main curve Cobb angle were performed in calibrated DICOM digital images using Surgimap® software.^20^ Flexibility index was calculated as postoperative correction divided by flexibility assessment (STR or FBR) correction in relation to the preoperative standing (IS) or sitting (NMS) radiograph as described previously^9,12,17^:

Flexibility index=(postoperative correction [%]/ flexibility assessment* correction [%])

Postoperative correction=[(Preoperative standing Cobb angle – postoperative standing Cobb angle) / (Preoperative standing Cobb angle)]ⅹ 100%

Flexibility assessment* correction=[(Preoperative standing Cobb angle – Preoperative supine bending Cobb angle) / (Preoperative standing Cobb angle)]ⅹ 100%

* supine traction radiograph or fulcrum bending radiograph

The flexibility index aims to display the relationship of the flexibility assessment and the postoperative correction with a value at or close to 1 showing a high congruence.

### Surgical Technique

The surgery was performed in a scoliosis center by 2 senior spine surgeons, experienced in scoliosis surgery. In all cases a dual rod system and anchoring pedicle screws were used, appropriately customized to the individual height of the patients and their pedicle diameter. The surgery was conducted with the patient in general anaesthesia in prone position with neuromonitoring using a triggered electromyogram device to allow for intraoperative measurement of sensory and motor evoked potentials. The strategic vertebral levels for anchoring of MCGRs were defined using preoperative standing radiographs of the patient as described before.^4^ A posteriormidline approach was established at the respective segments proximally and distally to the structural scoliotic curve. After dissection of the subcutaneous layer, the fascia was incised and a subfascial dissection was made along the spinous processes and the lamina vertebrae. The freehand technique based on specific anatomical landmarks was used to place the pedicle screws.^21^ The insertion of the rods was conducted through the proximal approach to the distal incision with a standard MCGR on the concave side and an offset MCGR on the convex side in antiparallel direction to prevent from impairing interaction of the magnetic components during later lengthening procedures. Intraoperative instrumental measurement of correction force was not conducted. After firm connection of the rods with the pedicle screw heads, the respective tissue layers were readapted, and the wound was closed. In all patients, neuromonitoring did not display impairment of evoked potentials and postoperative neurological impairment was not observed clinically.

### Data Management and Statistical Analysis

Baseline data as well as measurement data from image analyses were tabulated per group and were imported in to SPSS® software (IBM®, USA) for statistical analysis. For comparison between X-ray modalities within the total and subgroup populations, paired *t* test was used. When comparing the means of more than 2 groups, ANOVA was performed. For all analyses, significance level was set at *P* < 0.05.

## Results

For 50 patients that underwent the primary implantation of a MCGR implant between 2018 and 2022, preoperative STR as well as FBR were available. Within this population, 34 patients suffered from idiopathic scoliosis (IS) and 16 patients were diagnosed with neuromuscular scoliosis (NMS) (Table 1). The IS group contained a greater portion of female patients (82% vs 56%), which is in accordance to the known gender prevalence. In the NMS group, longer instrumentation lengths were used in average, as nonambulatory NMS patients received thoracopelvic instrumentation (Figure 1).

**Table 1.** Baseline Demographic Data.

	AIS (n = 34)		NMS (n = 16)		Total (n = 50)		
Parameter	n (%)	Mean (Range)	n (%)	Mean (Range)	n (%)	Mean (Range)	Mean Diff. (95% CI)
Female sex	28 (82)		9 (56)		37 (74)		n/a
Age at surgery		11.9 (3-16)		11.3 (4-14)		11.7 (3-16)	−0.6 (−2.07-0.86)
BMI		18.7 (13.2-29.1)		16.9 (12-26)		18.2 (12-29.1)	−1.83 (−4.5-0.85)
Instrumented vertebrae		12.1 (10-14)		15.2 (9-17)		13.1 (9-17)	n/a

Postoperative images of MCGR surgery displayed significant improvement of main curve Cobb angle (total: mean 38.0°, SD 13.1; IS: 36.0, SD 13.3; NMS: mean 42.4, SD 11.3) compared to preoperative images (total: mean 69.8°, SD 17.8, *P* < 0.001; IS: 66.9, SD 17.7, *P* < 0.001; NMS: mean 76.0, SD 16.3, *P* < 0.001). Further, for flexibility assessments significant main curve Cobb angle correction was observed: STR (total: mean 44.8°, SD 13.1, *P* < 0.001; IS: 42.4, SD 12.2, *P* < 0.001; NMS: mean 49.9, SD 13.6, *P* < 0.001) and FBR (total: mean 39.6°, SD 14.4, *P* < 0.001; IS: 37.3, SD 14.3, *P* < 0.001; NMS: mean 44.5, SD 13.3, *P* < 0.001).

**Figure 2.**
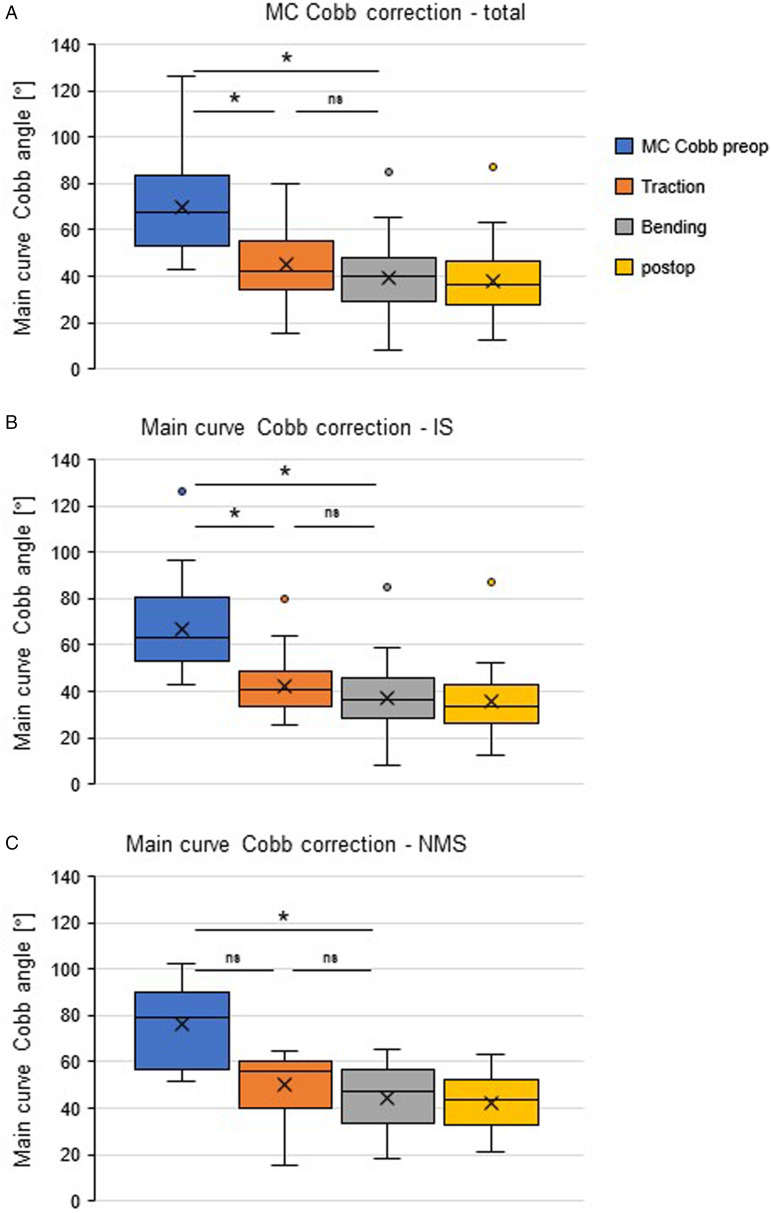
Correction of main curve Cobb angle. Displayed are the main curve cobb angles, analyzed in the indicated radiograph assessments for the total cohort (A) as well as IS (B) and NMS (C) subgroups. Quartiles, mean values (x), and mean values (line within box) are shown. The box covers the interquartile interval, the upper and lower side of the box represent the upper and lower quartiles. The whiskers end at the maximum and minimum value. The asterisk indicates statistical significance *P* < 0.05.

Furthermore, in comparison with postoperative radiographs, STR Cobb angles were found to be significantly higher in total (*P* < 0.0001) and both subgroups (IS: *P* = 0.0003; NMS: *P* = 0.0009), whereas for FBR no significant differences compared to postoperative Cobb angles were detected (total: *P* = 0.22; IS: *P* = 0.36; NMS: *P* = 0.44) (Figure 2).

**Figure 3.**
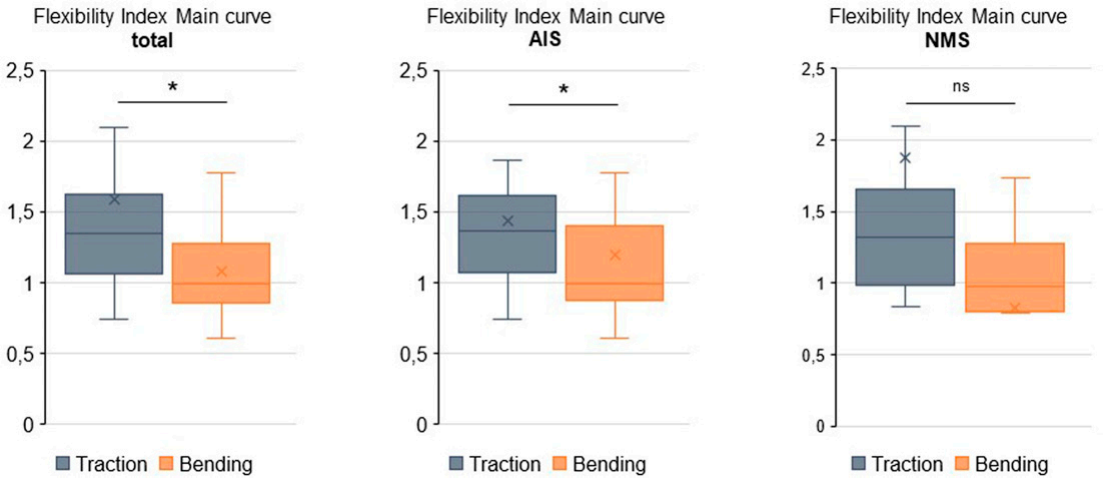
Flexibility indices. This comparative demonstrations show the fold change over postoperative correction as “flexibility index” (described above). Quartiles, mean values (x), and mean values (line within box) are shown. The box covers the interquartile interval, the upper and lower side of the box represent the upper and lower quartiles. The whiskers end at maximum and minimum values.

Calculation of flexibility index displayed a significantly lower congruence with postoperative correction according to higher flexibility indices for STR compared with FBR in total (1.58 vs 1.08; *P* = 0.024) and the IS subgroup (1.43 vs 1.19; *P* = 0.033) (Figure 3). In the NMS subgroup, no significant differences in flexibility index were detected between STR and FBR (1.88 vs 0.83; *P* = 0.164) (Figure 3).

## Discussion

To our best knowledge, this is the first study that evaluates the flexibility as well as correction potential of lateral bending and supine traction radiographs for young patients with juvenile idiopathic and neuromuscular scoliosis that underwent MCGR-implantation surgery.

Retrospective analysis of the preoperatively taken radiographs revealed a superior flexibility of the main curve for FBR in total and the IS subgroup. Moreover, in the total population as well as in the IS subgroup, STR showed significantly lower correction compared to postoperative X-rays. The most important finding in this study is, that FBR of the main curve displayed higher congruence with postoperative correction than STR in IS patients, whereas for NMS subgroup no statistical difference in flexibility index between STR and FBR was found. This reveals a good preoperative prediction of main curve correction of both flexibility assessments with high precision of FBR for IS patients. These findings are of notable relevance as the predictability of flexibility radiographs in MCGR surgery is yet undescribed.

Achieving appropriate initial correction in MCGR implantation is crucial as further correction during the process of distraction cannot be expected and many patients display vertebral autofusion within their period of rod lengthening.^18^ In contrast to posterior spinal fusion, MCGR is only anchored at the proximal and the distal end, whereas no pedicle screws are placed around the apex of the curve. It is therefore relevant to assess flexibility to predict the amount of correction which can be achieved as more rigid curves, predominantly rigid kyphoscoliosis, can hinder the implantation of the rods.

Further, in growth-accompanying treatment with MCGR and later definitive spinal fusion, the main correction of the deformity is already achieved through MCGR implantation. Hence, it is crucial to plan MCGR implantation with the same precision as the definitive spinal fusion as the instrumentation length with upper and lower instrumented vertebrae as well as the instrumented segments (mono- or bisegmental pedicle screw cranial instrumentation) are to be determined.

Moreover, better prediction of surgical correction and instrumentation length can help the surgeon in counseling the patient about the expected correction and to plan the respective surgical procedure. Hence, an adequate preoperative preparation and planning procedure is crucial and should be relying on scientific evidence.

In prior studies, for curves <50° Cobb angle, a greater flexibility was observed in bending images, whereas curves >60° were found to be more flexible in supine traction imaging technique.^7,15,16^ In comparison with active side bending radiographs, FBR previously were reported to show greater curve correction.^8,9,17^ Traction radiographs can be advantageous for neuromuscular scoliosis patients, were FBR are hard to perform. Additionally, traction radiographs also provide a lucid representation of the whole spine with structural and compensatory curves on 1 image.^13^ Moreover, in STR the rotation of the patient’s torso can be controlled better as in side-leaning FBR, where an undesired rotation could lead to underestimation of the curve in the planar representation.

For PSF in adolescent or young adult patients, a high correlation of the curve reduction achieved by traction radiographs and the postoperative correction as well as an equivalent correction potential compared to SBR was described.^22^ In moderate curves a higher flexibility and better prediction of postoperative correction was found for FBR compared to traction under general anesthesia and SBR.^23^ Two studies reported better flexibility for curves larger than 65° and higher prediction of the postoperative curve reduction for STR under general anesthesia compared to preoperatively taken FBR.^23,24^ However, 1 could argue that spine flexibility in a lateral bending position is most likely equivalently dependent from muscle tonus as in traction assessment. Thus, the comparability of the 2 imaging modalities might be low in these studies. Liu et al. investigated patients diagnosed with IS under general anesthesia and observed equivalent flexibility in STR and SBR.^25^ Moreover, preoperative planning and estimation of the curve correction usually takes place not immediately before surgery with patient already narcotized but earlier.

As both types of stress-imaging rely on good compliance of the patient, the results of curve correction show relatively high variation in our study.

In NMS patients, the correction potential of Traction and Bending flexibility assessment radiographs patients has barely been studied. We found 1 study that describes equivalent flexibility indices of preoperative STR and intraoperatively taken prone radiographs but higher flexibility of STR compared to preoperative supine radiographs.^26^ In a case series of 41 NMS patients, Bane and Luhmann proposed push-supine radiographs to predict coronal deformity correction of thoracopelvic PSF.^27^ We could not find comparative studies of traction and bending radiographs in NMS patients in the current literature.

Limitations of this study were the retrospective study design, which did not allow for a controlled setting and standardized process of the imaging procedure. Further, the low number of patients in the NMS group limits the statistical power of the study and could lead to an underestimation of potential differences between both imaging modalities. Hence, the herein presented data about the NMS group can only be descriptively analyzed. As MCGR for NMS is a rare indication and the underlying conditions are heterogenous, multicenter studies could provide adequate statistical power to address comparative research questions concerning preoperative flexibility radiographs.

## Conclusion

Side bending radiographs showed greater accordance with postoperative correction after primary MCGR implantation in IS patients compared to supine traction imaging and might therefore be the more precise tool to predict postoperative correction potential. However, traction radiographs also prevent good predictability with the advantage of a full spine image and combined assessment of all underlying curves.
